# Role of Nrf2 in Synaptic Plasticity and Memory in Alzheimer’s Disease

**DOI:** 10.3390/cells10081884

**Published:** 2021-07-25

**Authors:** Don A. Davies, Aida Adlimoghaddam, Benedict C. Albensi

**Affiliations:** 1 Division of Neurodegenerative Disorders, St. Boniface Hospital Research, Winnipeg, MB R2H 2A6, Canada; AAdlimoghaddam@sbrc.ca; 2 Department of Pharmacology & Therapeutics, University of Manitoba, Winnipeg, MB R3E 0T6, Canada

**Keywords:** NF-κB, neurodegeneration, oxidative stress, reactive oxygen species, inflammation

## Abstract

Nuclear factor erythroid 2-related factor 2 (Nrf2) is an important transcription factor that reduces oxidative stress. When reactive oxygen species (ROS) or reactive nitrogen species (RNS) are detected, Nrf2 translocates from the cytoplasm into the nucleus and binds to the antioxidant response element (ARE), which regulates the expression of antioxidant and anti-inflammatory genes. Nrf2 impairments are observed in the majority of neurodegenerative disorders, including Alzheimer’s disease (AD). The classic hallmarks of AD include β-amyloid (Aβ) plaques, and neurofibrillary tangles (NFTs). Oxidative stress is observed early in AD and is a novel therapeutic target for the treatment of AD. The nuclear translocation of Nrf2 is impaired in AD compared to controls. Increased oxidative stress is associated with impaired memory and synaptic plasticity. The administration of Nrf2 activators reverses memory and synaptic plasticity impairments in rodent models of AD. Therefore, Nrf2 activators are a potential novel therapeutic for neurodegenerative disorders including AD.

## 1. Introduction

Alzheimer’s disease (AD) is the most common cause of dementia and is the 6th leading cause of death in the United States (Centers for Disease Control and Prevention). There are close to 50 million individuals with AD globally, and ~6 million individuals in the United States alone (Alzheimer’s Disease International) [1,2]. Neuropathologically, AD is defined by the accumulation of senile plaques, largely composed of extracellular deposits of β-amyloid (Aβ) peptide, and neurofibrillary tangles (NFTs), composed of intracellular filamentous aggregates of hyperphosphorylated tau protein [3,4,5,6]. The time-dependent appearance of Aβ plaque deposits followed by NFTs are well-established hallmarks of AD, leading to synapse loss and neuronal death [7,8]. The time-dependent appearance of Aβ plaque deposits, followed by NFTs, leads to synapse loss and neuronal death. Many experimental drugs attempt to inhibit the formation of Aβ plaques and tau proteins or promote their disposal. Recently, aducanumab was approved to treat Alzheimer’s Disease by removing Aβ plaques in the brain. Aβ plaques do not always lead to AD, which raises concerns over the effectiveness of aducanumab. Other approved drugs for AD mask the symptoms of AD and do not provide disease modification. The majority of approved drugs for AD are acetylcholinesterase inhibitors, which increase the levels of acetylcholine by inhibiting the enzyme acetylcholinesterase that breaks down acetylcholine. Therefore, the investigation of novel therapeutic targets, such as regulating oxidative stress is of great importance to the development of novel AD treatments.

Oxidative stress can be considered an imbalance between free radicals and antioxidants. Oxidative stress is also an early observation in AD [9]. Free radicals are molecules with an unpaired electron in their outer orbit. The brain is highly susceptible to oxidative stress, given its high oxygen consumption and the high content of polyunsaturated fatty acids [10]. The excessive production of free radicals can result in the accumulation of β-amyloid (Aβ) and tau proteins, which are also hallmarks in AD [11]. At normal physiological concentrations, free radicals are necessary for synaptic plasticity and therefore learning and memory [12]. However, in neurodegenerative diseases, such as AD, when oxidative stress is increased, synaptic plasticity and memory are impaired [12]. Oxidative stress is widely studied as a therapeutic target to treat the learning and memory impairment in AD. Nuclear and mitochondrial levels of 8-Hydroxy-deoxyguanosine (8-OHdG), a biomarker of DNA oxidative damage, are elevated in AD patients and animal models of AD [13]. Mitochondrial dysfunction and inflammation are associated with oxidation of nucleic acids, protein, and lipids [14,15]. Copper is a metal involved with oxidative damage and associated with AD. However, copper can have beneficial effects depending on the metal delivery. In animal models, treatment with copper (II)-Bis (thiosemicarbazonato) improved cognition and decreased Aβ [16,17].

Nuclear factor erythroid 2-related factor 2 (Nrf2) is a key transcription factor that regulates oxidative stress with a basic-region leucine zipper (bZIP) in the cap-n-collar (CNC) family. In the absence of oxidative stress, Nrf2 is bound to its cytoplasmic inhibitor Kelch-like ECH-associating protein 1 (Keap1). Keap1 suppresses activation of Nrf2 by sequestering it in the cytoplasm, and by targeting it for ubiquitination [18,19,20,21]. In addition to functioning as an inhibitor of Nrf2, Keap1 also senses oxidants via its redox-sensitive cysteine residues [22,23,24]. Oxidative stress ends the inhibition of Nrf2 by Keap1 via an impairment of the ability of Keap1 to target Nrf2 for ubiquitination [25,26,27]. Nrf2 declines with age and the loss of Nrf2 allows unmitigated oxidative stress that drives age-related pathologies, such as a loss of proteostasis, genomic instability, telomere attrition, epigenetic alterations, cellular senescence, and mitochondrial dysfunction [28].

## 2. Nrf2 and its Role in Alzheimer’s Disease

Oxidative stress is involved with the occurrence and progression of AD. Aβ elevation is associated with increased levels of oxidation products from proteins, lipids and nucleic acids in the hippocampus and cortex of humans with AD [29]. In contrast, lower Aβ levels in the brain are correlated with lower oxidative stress markers [30]. Aβ plaques can reduce Ca^2+^ storage in the endoplasmic reticulum, which results in an excess of Ca^2+^ in the cytosol [31]. Due to the excess of cytosolic Ca^2+^, glutathione (GSH) levels are decreased and reactive oxygen species (ROS) can accumulate in the neurons [32]. The oxidative stress in AD patients may be a result of excitotoxicity from the glutamatergic *N*-methyl-d-aspartate (NMDA) receptors. NMDA receptor activation in AD has been shown to result in an excessive influx of Ca^2+^ by increasing cell permeability and generation of ROS and reactive nitrogen species [33,34]. In addition, Aβ can initiate free radical formation by activating NADPH oxidase [35]. Furthermore, abnormal aggregates composed of p-Tau protein lead to increased ROS production in AD. ROS was the key result of impaired axonal transport and caused by abnormal p-Tau protein [15].

**Figure 1:**
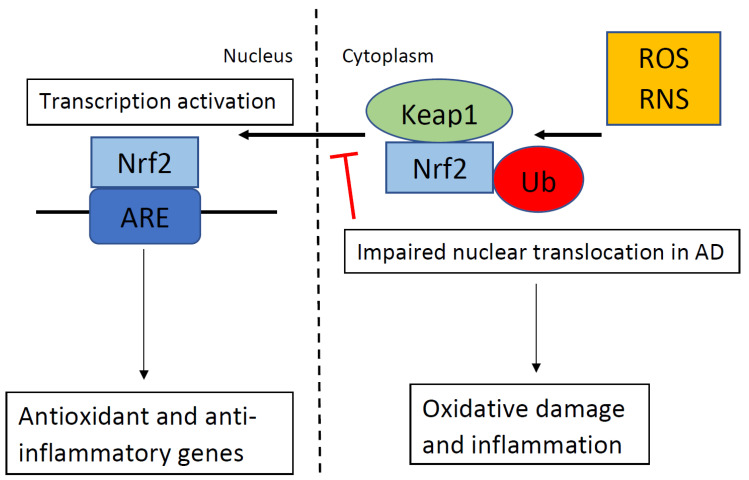
In the absence of oxidative stress. Kelch-like ECH-associating protein 1 (Keap1) suppresses the activation of nuclear factor erythroid 2-related factor 2 (Nrf2) and targets Nrf2 for ubiquitination (Ub). When Keap1 detects oxidative stress via reactive oxygen species (ROS) or reactive nitrogen species (RNS), as shown in the top right portion of the figure, Keap1 ends the inhibition of Nrf2, and Nrf2 translocates into the nucleus. Nrf2 binds to the antioxidant response element (ARE), which regulates the expression of antioxidant and anti-inflammatory genes. In Alzheimer’s disease (AD), nuclear translocation in response to ROS/RNS is impaired, which results in oxidative damage and inflammation.

Nrf2 is a key endogenous modulator in the protection against oxidative stress. In response to oxidative stress, Nrf2 translocates from the cytoplasm into the nucleus and transactivates genetic expression with antioxidant activity. AD patients had less nuclear Nrf2 in the CA1 region of their hippocampus than the controls despite oxidative stress markers in the hippocampal neurons of patients with AD [36]. This indicates that Nrf2 was not translocating from the cytoplasm into the nucleus in hippocampal neurons in patients with AD, despite oxidative stress markers in these neurons and an abundance of nuclear Nrf2 in the neurologically normal age matched controls (see Figure 1). Therefore, some process may be blocking Nrf2 nuclear activity, which may contribute to neuronal dysfunction. The levels of cytoplasmic Nrf2 are not different between age-matched controls and patients with AD. Albeit, the nuclear impairment is not the result of a general loss of Nrf2 protein but could reflect dysfunctional nuclear trafficking. Since the two hallmarks of AD are misfolded proteins, Aβ plaques and NFT, it is likely that endoplasmic reticulum stress is active in the hippocampus during the progression of AD, which may alter the Nrf2 pathway in the hippocampus. Methylene blue treatment in a mouse model of tauopathy increased the activation of Nrf2 and reduced tauopathy and oxidative stress [37]. Treatment with methylene blue was also associated with improved behavior with reduced locomotor abnormality, reduced anxiety abnormality, and improvement in learning and memory. Therefore, methylene blue may be a novel treatment option for people with AD because methylene blue reduces tau, which is one of the hallmarks of AD.

Initially, ROS was thought to only have negative physiological effects. However, others have observed the beneficial effects of ROS on mitochondria and in various cellular pathways [38,39]. Low levels of ROS are shown to have beneficial effects while high levels of ROS are associated with AD, suggesting a threshold determines whether ROS is beneficial or harmful [40]. The low levels of ROS regulate various cellular pathways, such as H_2_O_2_ regulating various signaling pathways with proteins containing cysteine residues [41]. Given the beneficial effects of low levels of ROS, Nrf2 activators should only be considered when ROS levels have crossed the threshold from beneficial into harmful.

## 3. Synaptic Plasticity and Reactive Oxygen Species

Synaptic plasticity is measured via the change of strength in synapses. Synaptic plasticity is associated with learning and memory [42]. Long term potentiation (LTP), a sustained increase in synaptic strength is associated with learning and memory. Many studies have been conducted to elucidate the effect ROS has in LTP. Superoxide and H_2_O_2_ are two forms of ROS that have effects on LTP. The treatment of hippocampal neurons with NMDA, AMPA, and kainic acid increases superoxide production [43]. NMDA receptor activation causes an influx of Ca^2+^ into the neurons, which is critical for most forms of LTP. Superoxide regulates the activities of the extracellular signal-regulated kinase (ERK) [44] and protein kinase C (PKC) [45]; both are essential for normal LTP. Both pharmacological blockage of NADPH oxidase and transgenic mice without NADPH oxidase proteins had impaired LTP [46]. Transgenic mice that overexpressed superoxide dismutase (SOD), a superoxide scavenger, had LTP impairment in mice overexpressing either the extracellular SOD (EC-SOD), or the SOD-1 isoforms [47,48]. There are three different SOD isoforms, SOD1 being cytosolic, SOD2 is mitochondrial, and SOD3 is the extracellular isoform [49,50]. Both SOD1 and SOD3 isoforms use copper and zinc as cofactors. The mechanism responsible for the LTP impairments are different between the SOD isoforms. Mice that overexpressed extracellular SOD had LTP impairment due to a reduction of superoxide in the hippocampus [51]. However, mice that overexpressed SOD-1 had an LTP deficit because of increased H_2_O_2_ production due to superoxide dismutation [47].

H_2_O_2_ has been shown to cross membranes via select aquaporin channels [52]. Aquaporin-4 is the predominant water channel in the central nervous system (CNS). LTP is impaired in transgenic mice with null aquaporin-4, which suggests that aquaporin channels are involved with synaptic plasticity [53]. However, these results do not show that H_2_O_2_ contributes to the impaired synaptic plasticity via aquaporin channels. In the CA1 region of the rat hippocampus, H_2_O_2_ inhibited LTP and caused a reduction in the population spike amplitudes and excitatory postsynaptic potential (EPSPs) parameters [54]. In contrast, H_2_O_2_ caused an increase in the activity of sympathetic preganglionic neurons [55]. These conflicting results may be due to the dose of H_2_O_2_ (mM in the Katsuki and colleagues’ study compared to nmol in Lin and colleagues’ study). H_2_O_2_ modulates LTP in a dose-dependent manner with 1 µM treatment increasing LTP, whereas 20 µM treatment did not affect the expression of established LTP [56].

## 4. Memory and Reactive Oxygen Species

ROS is a so-called double-edged sword with normal levels important for learning and memory, but increases in ROS resulting in impaired learning and memory. The Morris Water maze (MWM), which assesses spatial learning and memory has been used to examine the role of ROS in learning and memory. Over a number of trials, the rodent learns the position of a submerged platform in the MWM. Transgenic mice overexpressing SOD-1 took significantly longer to locate the hidden platform [57]. When the mice completed the test with a visible platform, both wild type and transgenic mice located the platform in equal time, suggesting that the overexpression of SOD-1 results in a spatial learning impairment rather than a perceptual or motor impairment. The spatial learning in the MWM is hippocampal dependent and as previously discussed SOD-1 transgenic mice have impaired LTP, suggesting superoxide is required for intact memory and synaptic plasticity [47]. SODs are involved with the dismutation of superoxide into H_2_O_2_.

EC-SOD mice have been examined on learning and memory behavioral tasks. The radial-arm maze assesses spatial memory and is shown to be dependent on the hippocampus. The radial-arm maze consists of eight arms, each of which is baited with a food reward. The choice accuracy measure is the number of correct arm entries before an error is made. Response latency is another measure that is assessed in the radial-arm maze. EC-SOD mice at 27 months and 30 months old had significantly higher choice accuracy levels than the control mice [58]. Therefore, EC-SOD overexpressing mice maintained their high levels of accuracy while the control mice had an aging-induced decline in accuracy. The response latency did not differ between the groups, suggesting that motor function did not contribute to the differences between the groups. In a fear-learning task, EC-SOD transgenic mice exhibit deficient hippocampal dependent associative memory. Contextual fear conditioning over a long retention interval was significantly impaired in EC-SOD transgenic mice [48].

Overexpression of SOD-2 in young or old mice does not affect memory in the MWM [59]. Consistent with these results, LTP was unaffected in mice overexpressing SOD-2. In addition to the regulatory role that ROS plays in memory and synaptic plasticity in neurologically normal brains, ROS has a pathological role in AD, as previously discussed.

## 5. The Role of Nrf2 in Synapse Plasticity

There is accumulating data, which shows an involvement of Nrf2 in synaptic plasticity. Nrf2 knock out mice have a deficiency in LTP of the perforant pathway in vivo [60,61]. Nrf2 knock out mice that combined amyloidopathy and tauopathy had impaired LTP in vivo [61]. In hippocampal slices, Nrf2 knock out mice had dysfunctional LTP [62]. Nrf2 knock out mice also exhibited decreased synaptic density and dendritic complexity [63]. Lipopolysaccharide (LPS) is a cell-wall immunostimulatory component of Gram-negative bacteria. The administration of LPS is frequently used to examine neuroinflammation-associated diseases in rodents. LPS injections are shown to impair LTP [64]. Nrf2 activators, including dimethyl fumarate and naringenin, decreased LPS toxicity [65,66]. APP/PS1 transgenic mice received the Nrf2 activator Dl-3-*n*-butylphthalide, which ameliorated synaptic plasticity deficits in an AD mouse model [67]. Linalool activates Nrf2, and was shown to reverse the decreased expression of synaptic plasticity-related proteins, including calcium-calmodulin-dependent protein kinase II (CaMKII), p-CaMKII, brain derived neurotrophic factor (BDNF), and tropomyosin-related kinase B (TrkB) in the hippocampus in an oxidative stress AD mouse model [68]. These studies demonstrate a link between the impairment of Nrf2 and the reduction of LTP. Furthermore, these studies show that the activation of Nrf2 is associated with the improvement of LTP.

**Figure 2:**
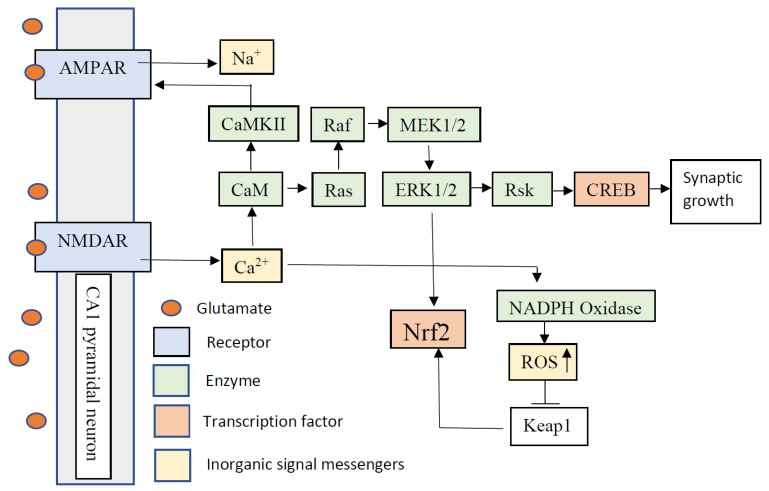
Putative Nrf2 signaling pathway during long-term potentiation (LTP) in the CA1 region of the hippocampus. Membrane depolarization and glutamate binding to the postsynaptic membrane receptors induce N-methyl-D-aspartate receptor (NMDAR) activation, and result in Ca^2+^ entry into the postsynaptic membrane. Ca^2+^ activates calmodulin (CaM) and calcium-calmodulin-dependent protein kinase II (CaMKII) and results in α-amino-3-hydroxy-5-methyl-4-isoxazolepropionic acid receptor (AMPAR) phosphorylation. When glutamate binds to AMPAR, they become permeable to Na+, which is important for stable activation of NMDAR. Ca^2+^ activates MAP kinase pathways (Ras/Raf/MEK1/2-ERK1/2-Rsk), which phosphorylate cAMP response element-binding protein (CREB). CREB induces mRNA transcription for synapse growth. Extracellular signal-regulated kinases (ERK1/2) can phosphorylate nuclear factor erythroid 2–related factor 2 (Nrf2). Keap1 is a negative regulator of Nrf2. Reactive oxygen species (ROS) inactivate Keap1, which allows Nrf2 to become active. NADPH oxidase is an ROS source that can be activated by Ca^2+^, and is translocated into neurons via NMDA receptors.

BDNF is a small dimeric protein that has high affinity binding with the tyrosine kinase, TrkB. BDNF and TrkB are broadly distributed across the subregions of the hippocampus. During late-LTP, protein synthesis occurs, which regulates the long-lasting changes in synaptic plasticity. BDNF is a key regulator in late-LTP [69]. High frequency stimulation results in BDNF secreted in a manner dependent on Ca^2+^ influx through NMDA subtype glutamate receptors or voltage-gated Ca^2+^ channels [70,71,72,73,74]. Lower hippocampal levels of BDNF in rats resulted in decreased nuclear translocation of Nrf2, leading to persistent oxidative stress [75]. The Nrf2 activator sulforaphane (SFN) prevented BDNF downregulation [76]. SFN induces nuclear translocation via its electrophilic property and regulates the phosphorylation of Nrf2 with different kinases [77]. In a triple transgenic mouse model of AD, SFN increased neuronal BDNF expression and increased levels of neuronal and synaptic plasticity molecules including microtubule-associated protein 2 (MAP2), synaptophysin, postsynaptic density protein 95 (PSD95), cAMP response element-binding protein (CREB), CaMKII, ERK, and Akt [78]. Short term fructose feeding, which results in an imbalanced redox homeostasis was associated with lower levels of BDNF, a lower amount of Nrf2, and decreases in the proteins involved with synaptic plasticity, including synaptophysin, synapsin I, and synaptotagmin I [79]. The GABA_A_ receptor antagonist gabazine and K^+^ channel antagonist 4-aminopyridine increased neuronal firing frequency and Ca^2+^ influx associated with the activation of synaptic NMDA receptors. This treatment activated Nrf2 signaling in cultured hippocampal cells [80]. When Nrf2 activation is induced by gabazine and 4-aminopyridine, it requires a developed synaptic network, including action potential firing, NMDA receptors, AMPA receptors, and metabotropic glutamate (mGlu) receptor activity. Nrf2 activation by gabazine and 4-aminopyridine was inhibited by blocking NMDA receptors, AMPA receptors and mGlu receptors [80]. These results show that glutamatergic receptors are important for regulating synaptic plasticity pathways. Figure 2 illustrates the signaling mechanism of the Nrf2 pathway during LTP.

## 6. The Role of Nrf2 in Memory

Various types of Nrf2 activators have been used to examine their effects on many different learning and memory tasks in rodents. The Nrf2 activators trans-cinnamaldehyde and curcumin reversed LPS-induced Aβ aggregation and the memory impairment in the MWM and in the novel object recognition task (NORT) [81]. The NORT measures nonspatial memory and takes advantage of the rodent’s innate preference for novelty. Rodents are allowed to explore identical items during the sample phase. After a delay period, the rodent is placed back into the area with one of the familiar objects replaced with a novel object. As rodents prefer novelty, memory is measured via the amount of time spent near the novel object compared to the time spent near the familiar object.

Carnosine is a dipeptide that is found in mammalian skeletal muscle and synthesized by carnosine synthase from the substrates beta-alanine and histidine [82]. Carnosine increased Nrf2 in the nucleus and improved memory in the Y-maze task [83]. The Y-maze task relies on the rodent’s innate preference to spontaneously alternate directions on each arm of the Y-maze. The rodent is placed at the end of one arm and allowed to move to the center where it can make a choice of which arm to go down. Rodents with intact memory will spontaneously alternate directions on each arm of the Y-maze whereas rodents with poor memory will perform the alternation at chance. 

Nrf2 activators improve cognition in preclinical rodent models of AD. Dietary supplementation with the Nrf2 activator anthocyanin improved memory of the MWM in a mouse model of AD [84]. The Nrf2 activator, ellagic acid, dose dependently improved memory in a rat model of AD [85]. In addition, expression levels of nuclear factor kappa B (NF-κB) in the hippocampus were increased with ellagic acid treatment. Ellagic acid treatment restored the nuclear/cytoplasmic ratio of Nrf2. Agmatine, an Nrf2 activator, improved memory in the MWM in a Streptozotocin-induced AD rat model [86]. Agmatine also suppressed the accumulation of Aβ and promoted the Nrf2-mediated antioxidant pathway. Quinovic acid improved memory on the MWM and Y-maze tasks in an Aβ mouse model of AD [87]. Moreover, quinovic acid downregulated phospho-NF-κB in the cortex and hippocampus. NXPZ-2 is a small-molecule compound that directly inhibits the Keap1-Nrf2 protein−protein interaction. NXPZ-2 treatment ameliorated learning and memory dysfunction in the Y-maze task in Aβ1–42-treated mice [88]. NXPZ-2 treatment increased serum Nrf2 and decreased serum Aβ1–42 levels in mice. Nrf2 deletion was associated with impaired memory in the MWM in APP/PS1 transgenic mice [89,90]. Aβ and p-tau were also increased in the hippocampus of APP/PS1 transgenic mice with an Nrf2 knockout. The Nrf2 activator coniferaldehyde reversed memory impairments in the MWM in APP/PS1 mice [91]. P-hydroxybenzyl alcohol has protective effects on Aβ-induced cell death and reversed memory impairments in the NORT and MWM [92]. Furthermore, p-hydroxybenzyl alcohol prevented decreased Nrf2 levels induced by Aβ_42_. The cocaine- and amphetamine-regulated transcript (CART) peptide is an extensively distributed neuropeptide in the (CNS) and is an activator of Nrf2. CART treatment improved spatial memory in the MWM task in rats infused with Aβ 1–42 into the hippocampus [93]. Mineralocorticoid receptor antagonists reduced memory impairment in the MWM task in mice with a brain infusion of Aβ 1–42 [94]. Mineralocorticoid receptor antagonists also activated the Nrf2-dependent antioxidant system. 

Peroxiredoxin 6 is an antioxidant protein that interacts with Nrf2. Peroxiredoxin is a major cellular antioxidant enzyme, but there are conflicting reports regarding the changes in activities of this enzyme in AD [95]. Mice overexpressing peroxiredoxin and infused with Aβ 1–42 had impaired memory on the MWM compared to Aβ 1–42 infusion alone and the control group [96]. Translocation of Nrf2 into the nucleus was increased in mice overexpressing peroxiredoxin with Aβ-infusions. Gracilins are sponge derived diterpenoid compounds that induce Nrf2 translocation. Conflicting results were obtained with a treatment of different gracilins in the MWM with one compound showing an improvement in memory and the other compound showing no effect on memory in 3 × Tg-AD mice [97]. These results may be due to the low sample size in the experiment. Treatment with sitagliptin and quercetin improved memory in the MWM in Aβ injected rats [98]. Additionally, the Nrf2 pathway was activated in brains of rats by sitagliptin and quercetin. Sitagliptin and quercetin treatment reduced the levels of Aβ in rat brains. Plumbagin protects against oxidative stress and inflammation by activating the Nrf2 pathway. Treatment with plumbagin, prior to streptozotocin brain infusions to model AD, prevented memory deficits in the MWM [99]. Inonotus obliquus polysaccharide reduces Keap1 levels, which enhances Nrf2 levels [100]. Treatment with inonotus obliquus polysaccharide in APP/PS1 transgenic mice improved memory in the MWM.

RTA-408, a covalent Keap1 inhibitor, is a potent activator of Nrf2, inhibitor of NF-κB and is in phase 2 for the treatment of mitochondrial myopathy [101,102]. Propofol decreases consciousness and memory and is used as a general anesthesia. RTA-408 protected against propofol-induced memory impairment assessed with the MWM and increased activation of Nrf2 and the inhibition of NF-κB p65 nuclear translocation [103]. Other studies have shown that RTA-408 increases Nrf2 and decreases NF-κB [104,105,106,107,108]. RTA-408 is a promising therapeutic for mitochondrial myopathy and immune disorders such as multiple sclerosis (MS) and Alzheimer’s disease.

Treatment with dimethyloxalylglycine in Aβ injected rats reversed memory impairment in the MWM and increased Nrf2 activation [109]. Activation of the Nrf2 pathway with SFN improved memory in the MWM in a mouse model of AD [110]. In contrast, blocking the Nrf2 pathway resulted in oxidative injury and decreased the cell viability of PS1V97L-Tg neurons. Antroquinonol is a ubiquinone derivative isolated from Antrodia camphorate and is shown to reduce oxidative stress and inflammatory cytokines via activating the Nrf2 pathway. Antroquinonol treatment for two months improved memory in the MWM and reduced hippocampal Aβ levels in APP transgenic mice [111]. Infusion of a viral vector expressing Nrf2 improved memory on the MWM in APP/PS1 mice [112]. Intranasal administration with a viral vector encoding human Nrf2 improved spatial memory impairment and diminished Aβ deposition in APP/PS1 mice [113]. Osthole is an extract from Cnidium monnieri fruits and is an anti-inflammatory agent that activates the Nrf2 pathway. APP/PS1 mice treated with osthole had improved memory on the MWM [114]. Carnosic acid is a proelectrophilic compound that is converted to its active form by oxidative stress, which stimulates the Nrf2 transcription pathway. Carnosic acid improved memory in the MWM in human amyloid precursor protein transgenic mice and 3 × Tg-AD mice [115]. Caffeic acid phenethyl ester is a natural bioactive compound found in many plants and activates Nrf2. Caffeic acid phenethyl ester treatment, after Aβ1–42 infusion, improved memory in the MWM but did not affect memory in the NORT [10]. Differences between the tasks may have contributed to the discrepant results such as the MWM depending on spatial cues in the environment and the NORT without environmental spatial cues. Kavalactone has been shown to reduce Aβ toxicity by inducing Nrf2 activity [116]. Kavalactone administration in APP/Psen1 mice reversed the memory impairments in the MWM [117]. Artemisinin is used as a malaria treatment and is an activator of the Nrf2 pathway. Artemisinin administered to 3 × Tg-AD mice improved memory on the MWM [118]. APP/PS1 transgenic mice received the Nrf2 activator Dl-3-n-butylphthalide, which improved memory in the NORT, MWM, and the Y-maze test [67].

Lifestyle can affect the expression of Nrf2 with diet and exercise contributing to Nrf2 expression in mouse models of AD. High-fat diet-induced obesity is a risk factor for AD. 3 × Tg-AD mice who had a high-fat diet had impaired memory in the MWM compared to 3 × Tg-AD mice who had a normal diet. The high-fat diet reduced the activation of Nrf2 by suppressing its upstream regulatory protein kinase B/Akt and the downstream targets such as heme oxygenase-1 and manganese SOD in mice. Exercise is shown to increase Nrf2 activity in rats. Exercise was also shown to improve memory in the NORT in a rat model of AD [119]. These studies suggest that lifestyle modifications such as diet and exercise influence Nrf2 expression. 

## 7. The Role and Regulation of NF-κB

Inflammatory mediators stimulate transcription factor NF-κB, which is a key regulator in the development of inflammation [120,121]. The NF-κB family of transcription factors is composed of several members such as: NF-κBp50/105, NF-κBp52/100 (RelB), NF-κBp65 (RelA), and NF-κBp75 (c-Rel) with dimers bound by the inhibitory protein IκB. NF-κB proteins are frequently located within the cytoplasm in an inactive state [122]. The activation of NF-κB involves canonical and noncanonical signaling pathways, which are critical for regulating immunity and inflammation; the activation of NF-κB has been well described [120,122,123]. In the canonical activation pathway, NF-κB p50, RelA and c-Rel become activated, whereas in the noncanonical pathway, NF-κB p52/100 (RelB) selectively becomes activated [124]. The canonical NF-κB pathway is stimulated through binding to innate and adaptive immune receptors such as: tumor necrosis factor receptor (TNFR), interlukin-1 receptor (IL-1R), toll-like receptors (TLR) ligand such as CD40L, and LPS. Binding with any one of these receptors activates the IκB kinase (IKK) trimeric complex (IKKα, IKKβ, and IKKγ (NEMO), which in turn leads to phosphorylation of IκBα, and subsequent degradation [124]. As a result, NF-κB p50/RelA dimers translocate from the cytoplasm into the nucleus, where they bind to the IκB site of chromosomes to regulate NF-κB-dependent targeted genes [123]. This differs from the noncanonical NF-κB pathway, which is triggered by a B-cell activating factor belonging to TNF family receptor (BAFFR), lymphotoxin β-receptor (LTβR), TNFR2, CD40L, and receptor activator for NF-κB (RANK). This binding, via several intermediate steps, leads to an interaction with IKKα, which then leads to the phosphorylation of NF-κB p100, and subsequently results in NF-κB p100 degradation. Once degraded, NF-κB p52/RelB dimers translocate into the nucleus to regulate and activate NF-κB targeted genes [124]. Transcriptional activity of NF-κB is also regulated by transcription coactivators and corepressors, such as CREB binding protein (CBP)/P300-associated factor, nuclear receptor corepressor, histone deacetylase (HDAC), p160 proteins (SRC-1, SRC-2, SRC-3), and SMRT. 

Short periods of inflammation in response to infection or tissue damage is beneficial. However, sustained inflammation can result in tissue injury and is associated with inflammatory diseases such as neurodegenerative disorders. NF-κB has various roles in innate immune cells including macrophages, dendritic cells and neutrophils. These innate immune cells express pattern recognition receptors (PRRs), which detect various microbes known as pathogen-associated molecular patterns (PAMPs). PAMPs are critical for the survival of the pathogen, and recognition of PAMPs by PRR result in antimicrobial immune activation via inflammatory cytokines and chemokines [125,126]. Necrotic cells release damage-associated molecular patterns (DAMPs), which PRRs detect [127]. PRRs are diverse and have specific properties that allow them to respond to different PAMPs and DAMPs. PRRs can activate the canonical NF-κB pathway and induce direct inflammation via chemokines and cytokines or indirectly via inflammatory T cells. Transforming growth factor-β-activated kinase 1 (TAK1) is involved with the signaling pathway of PRRs for NF-kB activation [128,129]. TAK1 can activate IKK, which regulates IκBα phosphorylation [130].

NF-κB regulates molecular pathways in adaptive immune cells, including CD4^+^ T-helper (Th) cells. RelA and C-Rel, which are two subunits in the canonical pathway of NF-κB, regulate T-cell receptor (TCR) and T-cell activation [131]. NF-κB mediates the regulatory T cell (Tregs) suppression of function via lack of 3-phosphoinositide-dependent kinase 1 (PDK1), which prevents NF-κB activation [131]. PDK1 regulates activation of PKC, which recruits and activates the IKK complex [132]. The noncanonical NF-κB pathway is necessary for differentiation and effector/memory of T cells [133,134]. Therefore, both the canonical and noncanonical NF-κB pathways are important for the regulation of T cells.

Accumulating studies have shown that the activity of NF-κB is significantly enhanced during the neurodegenerative process [123,135,136,137]. NF-κB subunits have been detected in neuronal and glial cells derived from AD brains [138]. Furthermore, a downstream target of NF-κB, transcription factor Nrf2, protect cells from various injuries via their antioxidant and anti-inflammatory effects, thus influencing the progression of disease [139]. Similar to NF-κB, Nrf2 is expressed in both neurons and glia [36,140]. It is reported that the NF-κB and Nrf2 signaling pathways regulate the expression of over 400 and 600 genes, respectively, that are associated with inflammation, neurodegenerative disorders, and other disorders [141,142]. An imbalance between the Nrf2 and NF-κB signaling pathways is associated with neurodegeneration [135,136]. Growing evidence suggests a cross-talk between the NF-κB and Nrf2 signaling pathways [143]. The molecular mechanisms underlining NF-κB/Nrf2 cross-talk depends on the cell type as well as the tissue context [144]. The complexity of Nrf2 and NF-κB cross-talk is discussed below.

## 8. Cross-Talk between the NF-ĸB and Nrf2 Signaling Pathways

Nrf2 signaling contributes to the anti-inflammatory process by regulating target genes via the antioxidant response element (ARE) and Keap1 system [145]. The Keap1/Nrf2/ARE signaling pathway mostly regulates the expression of anti-inflammatory genes and ultimately blocks the progression of inflammation [145]. Oxidative-stress mediated NF-κB activation can be blocked by the Keap1/Nrf2/ARE pathway [146,147]. NF-κB impacts the Keapl/Nrf2/ARE signaling pathway in three parts. First, Keap1 degrades IKK, which prevents the phosphorylation of NF-κB [146]. Second, oxidative stress, which activates IKK leads to phosphorylation of NF-κB and translocation of NF-κB from the cytoplasm into the nucleus stimulates the production of proinflammatory cytokines such as IL-1, IL-6, TNF-α, inducible nitric oxide synthase (iNOS), and cyclooxygenase-2 (COX-2) [148,149]. Ultimately, COX-2 reacts with Keap1 and activates Nrf2, which leads to the suppression of oxidative stress-mediated NF-κB activation [141]. Third, Nrf2 binds to CBP and other transcriptional machinery to begin ARE-driven gene transcription [150]. However, NF-κB inhibits Nrf2 activation by competing with Nrf2 for CBP and ultimately reducing ARE gene expression [150]. Overall, the Keap1/Nrf2/ARE signaling pathway inhibits the production of proinflammation [141]. Moreover, it has been demonstrated that Nrf2 directly regulates the expression of anti-inflammatory mediators such as CD36, IL-17D, macrophage receptor, and G protein-coupled receptor (GPCR) kinase, which suppresses the progression of inflammatory responses [151,152,153]. Nrf2 induces the anti-inflammatory phenotype of microglia and macrophages, while it decreases LPS-induced transcription of other NF-κB target genes [154,155]. Nrf2 increases cysteine and GSH levels in macrophages. However, depletion of GSH triggers macrophages to Nrf2 activation by LPS [156]. These findings show that Nrf2 acts as an anti-inflammatory marker, which is critical for regulating inflammatory responses.

## 9. Anti-Inflammatory Role of the Nrf2/HO-1 Signaling Pathway

From a functional perspective, Nrf2 negatively regulates oxidative-stress mediated NF-κB activation through the hemoxygenase-1 (HO-1) pathway [147]. Under normal physiological conditions, Nrf2 is bound to Keap1 in the cytosol. Under oxidative stress conditions, the sulfhydryl groups on Keap1 are oxidized, which changes the Keap1 configuration and releases Nrf2. Thus, Nrf2 translocates from the cytoplasm into the nucleus, which then binds with ARE genes such as HO-1 along with small musculoaponeurotic fibrosarcoma (Maf) proteins [147]. HO-1 is an essential enzyme involved in Nrf2-mediated NF-κB inhibition. Overexpression of HO-1 blocks IκB-α degradation, which inhibits NF-κB activity. HO-1 also inhibits the TNF-α-dependent activation of NF-κB [157]. Conversely, cells over-expressing NF-κB showed reduced HO-1, which confirms that NF-κB activation can act as an Nrf2 repressor [158]. HO-1 exerts anti-inflammatory functions via the production of carbon monoxide (CO). HO-1 catalyzes the heme into iron, CO, and bilirubin. CO acts as an NF-κB inhibitor, which leads to the reduced production of pro-inflammatory responses. Overall, the Nrf2/HO-1 pathway directly inhibits proinflammatory cytokines and activates anti-inflammatory cytokines [159]. These findings further suggest that the Nrf2 directed increase in the expression of HO-1 is crucial for cross-talk between Nrf2 and NF-κB. Furthermore, NF-κB increases the recruitment of histone deacetylase3 (HDAC3) to the ARE region by binding to Maf proteins and therefore interferes with the Nrf2 transcription factor [143].

## 10. Nrf2 Activating Compounds

Dihydroquercetin (DHQ) treatment significantly inhibited the upregulation of TNF-α, interferon-γ (IFN-γ), and TLR-4 after LPS stimulation in a macrophage-like cell line derived from BALB/c mice [160]. Additionally, DHQ mediates LPS-mediated anti-inflammatory responses via the Nrf2/HO-1 pathway in these cells. Taurine chloramine (TauCl) upregulates the Nrf2/HO-1 pathway, which reduces the expression of pro-inflammatory cytokines (e.g., IL-1β, IL-6, TNF- TNF-α), inhibition of phosphorylation and translocation of NF-κB in hippocampal H22 neurons and mouse BV2 microglia [161,162]. These outcomes suggest that TauCl is neuroprotective initiated by neuroinflammation. Furthermore, the Nrf2 activator, Nardochinoid C (DC), activates the Nrf2/HO-1 signaling pathway to inhibit inflammation and oxidative stress to contribute to its anti-inflammatory and antioxidant effects, which were inhibited by the Nrf2 siRNA and HO-1 blocker [163]. This suggests that the Nrf2/HO-1 axis plays a major role in anti-inflammatory functions and activation of Nrf2 is a potential treatment for prevention of diseases linked to inflammation and oxidative stress. Both in vitro and ex vivo data show that flavokawain A (FKA) remarkably suppressed the proinflammatory cytokine and increased the level of anti-inflammatory cytokine in BALB/c mouse-derived primary splenocytes [164]. FKA induced the HO-1 expression by increasing Nrf2 translocation into the nucleus via the Nrf2/ARE signaling pathway in these cells. Additionally, FKA treatment significantly downregulated the LPS-induced ROS production and blocked NF-κB activity [164]. Both in vitro and in vivo studies revealed that the active form of an electrophilic compound such as carnosic acid (CA) activates the Keap1/Nrf2 pathway, which provides neuroprotection benefits in AD models [115]. Yoshida et al., reported that CA reduced levels of Aβ42 in a neural cell line [165]. Additionally, upregulation of Nrf2 improved neuroprotection against Aβ neurotoxicity in an AD mouse model [89]. Further activation of Nrf2 reduced the level of phospho-tau protein [166]. The expression pattern of Nrf2 was completely distorted in the hippocampal neurons of AD, PD, and Lewy body dementia [36]. Another study showed that Nrf2 activation via triterpenoids decreased inflammation, oxidative stress, and memory deficits in AD mice. These findings revealed a clear link between Nrf2 and AD-mediated cognitive decline. A previous study revealed that Nrf2 activity is low in human neurons [167]. Further studies revealed that the beneficial effect of an Nrf2 injection in the CNS was based on the actions of astroglia [140]. Nrf2 activation can diminish the activity of microglia [168]. A deficiency of Nrf2 was associated with enhanced microglial responses in hippocampal tissue of AD mouse models [89]. Additionally, reduced microglial activity was revealed in an ALS mouse model when crossed with glial fibrillary acidic protein (GFAP)-Nrf2 mice [169]. These findings display an opposite connection between Nrf2 and microglial activation, thus supporting the concept that Nrf2 regulates neuroinflammation. Another study revealed that Nrf2 ^−/−^ mice did not express HO-1 in microglia, which led to increased microgliosis PD [168]. This finding demonstrates that Nrf2 modulates microglia dynamics in neurodegenerative disorders.

Deletion of Nrf2 has been linked with enhanced inflammatory responses, which is regulated by NF-κB [170]. Astroglia and microglia treated with LPS displayed upregulated NF-κB activity compared with nontreated cells. Thus, activated NF-κB enhanced the cytokine production that contributes to astrogliosis and neuronal loss, which are a cause of the neurodegenerative phenotype [171]. Nrf2 has the potential to reduce numbers of hyperactive astroglia and microglia, which actively contribute to the pathology of the CNS. Therefore, the risk of neurodegeneration arises when the balance between Nrf2 and NF-κB signaling pathways is disrupted. More specifically, a decline in Nrf2 activity and increase in NF-κB activity can lead to neuroinflammation and increased oxidative stress. Neuroinflammation due to upregulated NF-κB can activate astroglia and microglia, which further increases the production of proinflammatory cytokines [149]. A lack of Nrf2 is associated with enhanced IKKβ activity, which increases the phosphorylation of IκBα and its subsequent degradation leading to the activation of NF-κB [172]. Deletion of Nrf2 triggers the activity of NF-κB, thus elevating the cytokine production that is associated with astrogliosis and neuronal death, which is an underlying cause of neurological deficits. Both in vitro and in vivo studies revealed activation of Nrf2 decreased oxidative stress, neuronal apoptosis, and inflammatory responses through the blockage of the ROS/NF-κB pathway.

Further, Nrf2^−/−^ mice treated with LPS and TNFα displayed downregulated IKK activity, which led to increased phosphorylation and degradation of IκB [173]. This finding suggested that the NF-κB response is subsequently activated when Nrf2 is suppressed. Several studies have demonstrated that Nrf2 upregulates the expression of HO-1, which leads to the inhibition of NF-κB signaling that protects cells from H_2_O_2_ cytotoxicity [174,175].

Due to oxidative stress and inflammation, the expression and activity of Nrf2 is significantly decreased in neurodegenerative disorders. In fact, several chemopreventive agents have been identified as Nrf2 activators such as curcumin, phenethyl isothiocyanate (PEITC), and SFN, which significantly inhibited LPS-induced NF-κB activation in human colon HT-29 cancer cells [176]. Further, PEITC and SFN treatment in human prostate cancer PC-3 cells inhibits both phosphorylation of IKK/IκB and nuclear translocation of NF-κB p65 subunit, which suppresses the NF-κB signaling pathway [177]. SFN activates Nrf2 through activating the Keap1/Nrf2/ARE pathway and inhibiting NF-κB signaling [178,179]. Additionally, SFN alters mitochondrial dynamic proteins through Nrf2 mechanisms [180]. It has also been demonstrated that SFN inhibited TNFα-induced NF-κB activation through inhibiting IkB-α phosphorylation [181]. SFN revealed neuroprotective effects in both in vitro and in vivo models of AD, TBI, PD, MS, and stroke [178,182,183,184,185,186]. Curcumin, as a natural polyphenol compound, activates the Nrf2 signaling pathway through repressing inflammatory and electrophilic modification of KEAP1 [187]. Another study showed that curcumin protects brain cells from Aβ pathology and synaptic degradation, while enhancing spatial learning in AD mouse models [188]. Curcumin treatment reduces the expression of proinflammatory markers by inhibiting NF-κB activity in microglia [189]. Additionally, curcumin treatment downregulated the expression of NF-κB that leads to an elevation of Nrf2 activity and thus reduced neurological dysfunction in a rat model of cerebral ischemia and reperfusion [190]. Epigallocatechin gallate (EGCG) is another potent Nrf2 activator that has been shown to block the activity of NF-κB, and reduce Aβ plaques and memory decline [191,192]. Additionally, EGCG has shown beneficial effects on PD, MS, and TBI models via augmentation of Nrf2 activity and reduction of inflammatory responses [193,194].

Another Nrf2 activator, isoquercetin, upregulates antioxidant genes, attenuates inflammatory responses and regulates the NF-κB pathway in diabetic rats [195]. The neuroprotective effects of isoquercetin have also been reported in in vitro and in vivo cerebral ischemia/reperfusion injury models accompanied by increased Nrf2 activity and reduced inflammatory responses by inhibiting NF-κB activation [196]. Other neuroprotective medications are isovitexin and sappanone that exhibit anti-inflammatory effects through activation of the Nrf2/HO-1 pathway and inactivation of the NF-κB signaling pathway [197,198]. Moreover, eriodictyol displays an anti-inflammatory function by activating Nrf2 and inactivating NF-κB, which inhibits the expression of cytokines in macrophages [199].

Previous studies have shown that some pharmacological compounds such as melatonin and resveratrol suppressed the activity of NF-κB and activated Nrf2 signaling in an experimental diabetic neuropathy model. Treatment with melatonin and resveratrol increased the levels of Nrf2/HO-1, which further reduced the expression of proinflammatory markers such as IL-6, TNF-α levels, decreased expression of iNOS, COX-2, and reduced apoptosis [200,201].

## 11. Nrf2 and NF-κB Crosstalk with Other Transcription Factors

In addition to the cross talk between Nrf2 and NF-κB, there is cross talk among other immunomodulator transcription factors. Hypoxia-inducible factor 1 (HIF-1) is a heterodimeric transcription factor involved with the response to hypoxia and metabolism. HIF-1α is highly expressed in most innate and adaptive immune cells [202]. The HIF-1 and Nrf2 pathways are mediated by ROS and have many overlapping cellular pathways including vascular endothelial growth factor (VEGF), erythropoietin (EPO), and angiopoietin 2 (ANGPT2) [203]. The blockade of Nrf2 is associated with lowered HIF-1α at the post-translational level suggesting that Nrf2 is involved with the modulation of the prolyl hydroxylase domain containing proteins (PHDs) [204,205]. The Nrf2 genes are able to increase HIF-1 signaling, which may have resulted in poor colorectal cancer patient survival [206]. Hypoxic cellular conditions results in NF-κB activation in phagocytes, which activates HIF-1α [207,208,209]. HIF-1α increases neutrophil survival via activation of the NF-κB pathway, which results in persistent inflammation [210].

Activator protein 1 (AP-1) is a family of bZIP transcription factors consisting of two families of genes, Fos (c-Fos, FosB, Fra1, and Fra2) and Jun (c-Jun, JunB, and JunD) [211,212]. There is cross talk between AP-1 and Nrf2 with AP-1 activation decreased from the Nrf2 activators SFN and EGCG [213]. NF-κB and AP-1 transcription factors are modulated via different mechanisms. However, they are both activated with many of the same stimuli [214]. Many genes are required for the coactivation of AP-1 and NF-κB, which suggests they are working together [215].

The signal transducer and activator of transcription 3 (STAT3) is a transcription factor involved with inflammation. Activation of Nrf2 increased the levels of small heterodimer proteins (SHP) resulting in STAT3 repression [216]. The STAT3-NF-κB complex in the fascin promoter contributes to transcription when exposed to IL-6 and TNF-α [217]. The nuclear factor of activated T cells (NFAT) is a family of transcription factors involved with immune response. The pathways between NFAT, Nrf2, and NF-κB interact on several regulatory steps and are involved with tumor development and chemoresistance in pancreatic cancer [218]. NF-κB and NFAT share similar DNA binding domains and fast nuclear translocation when activated [219]. FOXO are a group of the Forkhead family of transcription factors that have conserved DNA binding domains and have a key role in immunoregulation. The activation of FOXO via ROS results in gene expression for antioxidants and might attenuate the activity of Nrf2 [220]. The primary FOXO member, FOXO3a inhibits NF-κB activation via Th activation [221].

## 12. Conclusions

More research is required to investigate the potential linkage between two crucial transcription factors, Nrf2 and NF-κB in neurodegenerative diseases. It is believed that investigating this linkage will greatly assist in developing therapeutic choices for slowing and/or preventing the onset of neurodegenerative disorders such as AD; it will also assist in preventing memory and synaptic plasticity impairments.

## Data Availability

Not applicable.
